# Whole-genome sequencing identifies persistent transmission of a high-risk ST2 *Acinetobacter baumannii* clone in a Guangzhou Hospital

**DOI:** 10.3389/fmicb.2026.1730485

**Published:** 2026-02-18

**Authors:** Xuxia Cui, Chenglong Lin, Wenjie Zhu, Xinqiang Zhang, Weisha Wang, Long Ye, Weijiang Liu, Suling Liu

**Affiliations:** 1 Laboratory Medicine, Guangdong Provincial People's Hospital (Guangdong Academy of Medical Sciences), Southern Medical University, Guangzhou, Guangdong, China; 2 School of Biomedical Engineering (Suzhou), Division of Life Sciences and Medicine, University of Science and Technology of China, Hefei, China; 3 CAS Key Laboratory of Biomedical Diagnostics, Suzhou Institute of Biomedical Engineering and Technology, Chinese Academy of Sciences, Suzhou, China

**Keywords:** *Acinetobacter baumannii*, genomic characteristics, molecular epidemiology, multidrug resistance, ST2, virulence factors

## Abstract

**Background:**

*Acinetobacter baumannii* (*A. baumannii*) has posed a serious threat to the global healthcare environment due to its widespread multidrug resistance. However, the long-term molecular epidemiological characteristics, drug resistance profiles and genomic characteristics of *A. baumannii* isolates in Guangzhou, China have not been fully elucidated. This study aims to systematically analyze these characteristics using *Acinetobacter baumannii* strains from a local tertiary hospital.

**Methods:**

A total of 98 non-repeating clinical isolates of *A. baumannii* collected between 2013 and 2021 were analyzed in the study. Whole genome sequencing technology (Illumina NovaSeq 6,000 platform) was also used for multi-locus sequence typing (MLST), resistance genomic/virulence genomic analysis (based on the CARD/VFDB database), plasmid screening (with the PlasmidFinder tool), and pan-genomic analysis (via the Roary tool).

**Results:**

Among the 21 identified STs, ST2 was the dominant lineage, accounting for 66.3% (65/98) of all isolates, indicating the establishment of a predominant epidemic clone. Compared with non-ST2 strains, ST2 isolates exhibited a significantly higher rate of carbapenem resistance (95.38%) and carried a higher burden of resistance determinants, including *bla*_OXA-23_, *bla*_ADC-25_, *tet(B)*, and multiple aminoglycoside resistance genes. Notably, ST2 strains harbored a highly conserved and dominant repertoire of virulence factors, particularly those involved in iron acquisition and host adaptation, such as *ompA*, *abaI*, and the complete siderophore synthesis and uptake systems (*basA*–*basJ*, *bauA*–*bauF*, and *entE*). These features likely confer enhanced survival, persistence, and transmissibility in the hospital environment, supporting the classification of ST2 as a high-risk epidemic clone. Consistent with this, genomic clustering and temporal aggregation of ST2 isolates suggested sustained intrahospital transmission during the study period. Pangenome analysis revealed that *A. baumannii* possesses a large accessory genome (76.4%), reflecting substantial genomic plasticity that may facilitate rapid adaptation to antimicrobial and host-derived selective pressures.

**Discussion:**

As the first long-term genomic epidemiological study of *A. baumannii* in Guangzhou, our findings confirm that ST2 is the predominant multidrug-resistant and outbreak-prone lineage, driven by the convergence of resistance gene accumulation, virulence optimization, and genomic flexibility. These results underscore the urgent need to strengthen infection control measures and antimicrobial stewardship to curb the continued spread of this high-risk clone.

## 1 Introduction

*Acinetobacter baumannii* is a major pathogen that causes hospital-acquired infections in China. As an obligate aerobic Gram-negative bacterium, it is widely distributed in healthcare settings and readily colonizes sites such as the skin, conjunctiva, oral cavity, respiratory tract, gastrointestinal tract, and urogenital tract of hospitalized patients (Luo et al., 2025). Infection with *A. baumannii* has been shown to lead to a range of complications, including ventilator-associated pneumonia, wound and urinary tract infections, and bacteremia. These further exacerbate the physical burden on patients who are hospitalised (Bian et al., 2021).

Carbapenem-resistant *A. baumannii* has emerged as a primary pathogen of global concern in the domain of hospital infection control (Hujer et al., 2005). Research indicates that approximately 40% of *A. baumannii* isolates demonstrated carbapenem resistance in 2021 (Jachowicz-Matczak et al., 2025). In the context of intensive care unit (ICU) settings, the implementation of pathogen surveillance has yielded noteworthy insights into the prevalence of carbapenem-resistant strains of *A. baumannii*. A comprehensive analysis of 148 isolates revealed a staggering 96.7% confirmation rate, with a significant 90% exhibiting extensively drug-resistant (XDR) characteristics (Ababneh et al., 2025). The emergence of multidrug-resistant strains has been shown to have a significant impact on the complexity of clinical treatment and the risk of therapeutic failure (Falagas et al., 2006).

ST2 (international clone IC2) represents a globally disseminated multidrug-resistant lineage and has been consistently reported as a predominant clone in hospital-acquired infections in China. A nationwide genomic analysis covering 27 provinces/municipalities from 1999 to 2022 demonstrated that ST2 accounted for 87.5% of clinical isolates, highlighting its long-term widespread circulation and sustained transmission advantage in the Chinese healthcare setting (Zhang et al., 2025).

Whole-genome sequencing (WGS) has superseded conventional epidemiological investigation methodologies in public health, evolving into a pivotal instrument for disease surveillance and outbreak response (Gorrie et al., 2022). The utilisation of WGS facilitates comprehensive and precise analysis of pathogen genome sequences, expeditious identification of outbreak origins, transmission pathways, and evolutionary trends. This, in turn, provides a robust foundation for the development of effective control strategies. The analysis of WGS data holds significant potential for applications in bacterial classification and the inference of epidemiological features. Specifically, by analysing specific genes within bacterial genomes, it is possible to accurately classify bacterial isolates and further infer their epidemiological characteristics, such as species identification, the detection of resistance genes, and the screening of virulence genes. Research indicates that proactive whole-genome sequencing of healthcare-associated bacterial pathogens significantly reduces morbidity and mortality while generating substantial cost savings for healthcare institutions, thereby offsetting the expenses of implementing genomic surveillance (Fox et al., 2023). The successful dissemination of *A. baumannii* is closely associated with its high genetic plasticity, which enables long-term persistence in hospital environments and the continuous accumulation of resistance- and virulence-associated genetic traits, thereby facilitating the sustained spread and outbreak formation of high-risk clones (Yehya et al., 2025). As technology advances, the scope and precision of WGS applications continues to expand (Quainoo et al., 2017). The present study employs whole-genome sequencing of *A. baumannii* to identify the emergence of novel virulence or resistance genes. Phylogenetic analysis is further employed to substantiate the occurrence of outbreaks involving homologous strains.

The objective of this study was to utilise whole-genome sequencing as a means to elucidate the molecular characteristics, antimicrobial resistance profiles, virulence determinants, and phylogenetic relationships of these isolates. The findings of the present study provide critical insights into the long-term dissemination and evolution of *A. baumannii* in this region, with implications for infection control and antimicrobial stewardship strategies.

## 2 Materials and methods

### 2.1 Bacterial isolation, genomic DNA extraction

A total of 102 *A. baumannii* isolates were obtained from the clinical microbiology laboratory of a tertiary hospital in Guangzhou, China, between 2013 and 2021. To preserve strain diversity and avoid the selection of genetically related isolates, a random sampling strategy was adopted, which took into account both the isolation month and sample source of the strains. Four isolates were subsequently identified as non-baumannii members of the *Acinetobacter calcoaceticus–baumannii* (Acb) complex and were excluded. The remaining 98 *A. baumannii* isolates were included in this study. These *A. baumannii* isolates were derived from multiple clinical specimens, including sputum (*n* = 54), bronchial irrigation fluid (*n* = 10), clean midstream urine (*n* = 5), wound exudate (*n* = 6), blood (*n* = 6), and bronchoalveolar lavage fluid (*n* = 3). Additionally, several other isolates were recovered from diverse sources such as cerebrospinal fluid, surgical resection tissues, and pleural effusion, among others. This study was reviewed and approved by the Ethics Committee of Guangdong Provincial People’s Hospital (Approval No. KY2023-1051-01).

*A. baumannii* strains were cultured in Luria-Bertani Broth, and stock cultures were stored in Luria-Bertani Broth supplemented with 25% (vol/vol) glycerol at −80 °C. Bacterial genomic DNA was extracted using the TIANGEN DNA Kit (TIANGEN, China) following the manufacturer’s recommended protocol, consistent with previous descriptions (Zhao et al., 2022). The quality and concentration of the extracted DNA were evaluated using a Nanodrop spectrophotometer (Micro-volume UV–Vis Spectrophotometer FC2100, China). The purified DNA samples were kept at −20 °C until subsequent experimental use.

### 2.2 Clinical data

**Figure 1:**
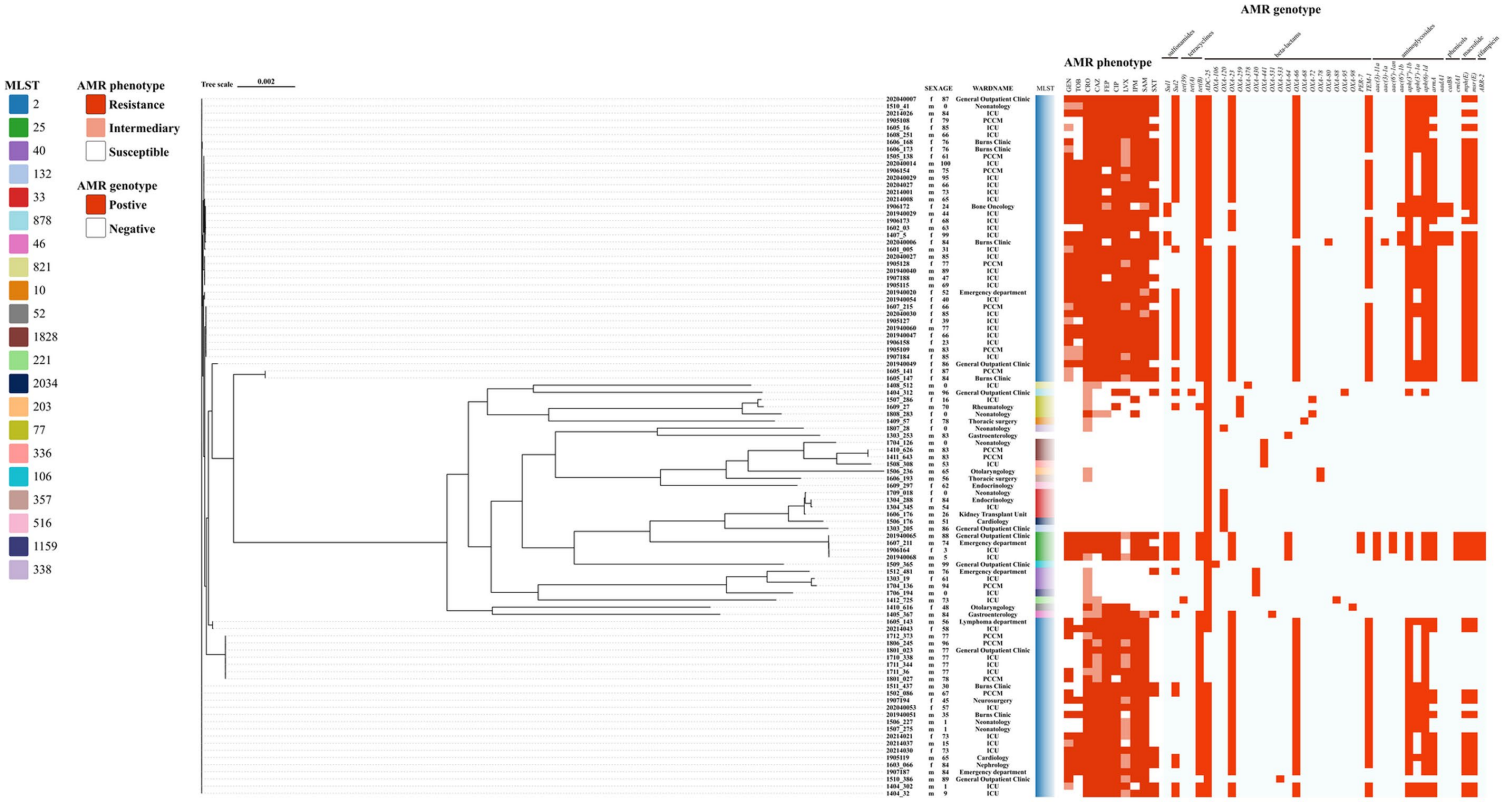
Genes mediating *A. baumannii* MLST typing and drug resistance (AMR phenotype and AMR genotype). MLST color code: isolates are colored by sequence type (ST) as indicated in the color key on the left. AMR phenotype: red, resistant; light orange, intermediate; white, susceptible. AMR genotype: red, gene present; white, gene absent. The first two digits of the strain name indicate the year of isolation; for example, strain 1710_338 was isolated in 2017.

In the present research, a retrospective analysis was conducted on the clinical data of 98 patients diagnosed with *A. baumannii* infection, and statistical processing was further performed on the relevant information of these patients. Beyond collecting demographic data—including indicators like age, gender, and underlying medical history—the investigation also gathered details related to hospitalization characteristics, such as the source of clinical samples and the department where the pathogen was isolated, with specific data presented in Figure 1.

### 2.3 Antimicrobial susceptibility testing

The antimicrobial susceptibility of *A. baumannii* isolates was determined using the VITEK-2 Compact automated system (bioMérieux, France), A*. baumannii* susceptibility testing of *Acinetobacter baumannii* was performed using the VITEK^®^ 2 AST-N335 antimicrobial susceptibility testing card (bioMérieux, Marcy-l’Étoile, France). When required, supplementary antimicrobial susceptibility testing was performed using the disk diffusion method from Oxoid (Thermo Fisher Scientific, Basingstoke, UK). Susceptibility results were interpreted according to the Clinical and Laboratory Standards Institute (CLSI) M100 guidelines corresponding to the year of isolate collection.

The tested antimicrobial agents included amikacin (AMK), gentamicin (GEN), tobramycin (TOB), ceftriaxone (CRO), ceftazidime (CAZ), cefepime (FEP), ciprofloxacin (CIP), levofloxacin (LVX), imipenem (IPM), meropenem (MEM), ampicillin/sulbactam (SAM), piperacillin/tazobactam (TZP), cefoperazone/sulbactam (SCF), and trimethoprim–sulfamethoxazole (SXT). For cefoperazone/sulbactam, CLSI cefoperazone breakpoints were applied. Quality control was performed using *Staphylococcus aureus* ATCC 29213 and ATCC 25923, and *Escherichia coli* ATCC 25922, as recommended by the National Center for Clinical Laboratories of China.

### 2.4 Whole genome sequencing and resistome and virulome characterization

Comprehensive genomic profiling was carried out for the entire set of 98 *A. baumannii* isolates. Libraries with ~350 bp inserts were prepared and sequenced on the Illumina NovaSeq 6,000 platform; the resulting high-quality reads were then *de novo* assembled with SPAdes v3.15.3. Antibiotic-resistance determinants were annotated by querying the CARD database under criteria of ≥80% nucleotide identity and ≥80% sequence coverage (CARD),^1^ whereas virulence factors were retrieved through parallel screening of the VFDB with 80% identity and 80% query-coverage cut-off values (VFDB).^2^

### 2.5 Whole genome sequencing analysis

Whole-genome sequencing was performed for all 98 *A. baumannii* isolates. Genomic DNA was extracted, and sequencing libraries were prepared using the Illumina DNA Prep Kit (Illumina, San Diego, CA, USA) according to the manufacturer’s instructions. Paired-end sequencing (2 × 150 bp) was conducted on the Illumina NovaSeq 6,000 platform, generating libraries with an average insert size of approximately 350 bp. High-quality reads were obtained after quality control and trimming, and *de novo* assembly was performed using SPAdes v3.15.3 (Antipov et al., 2016). Antibiotic resistance genes were identified by querying the assembled genomes against the Comprehensive Antibiotic Resistance Database (CARD) using thresholds of ≥80% nucleotide identity and ≥80% sequence coverage. Virulence factors were predicted by parallel screening against the Virulence Factors Database (VFDB)^3^ using the same cut-off values (≥80% identity and ≥80% coverage).

Using the first ST2 isolate collected in 2014 (1404_302) as the reference genome, core-genome single-nucleotide polymorphism (SNP) analysis was performed for all ST2 isolates to assess their genetic relatedness and to identify potential transmission clusters. High-quality SNPs from all isolates were combined to generate a core SNP alignment, and putative recombinant regions were detected and masked using Gubbins to minimize the impact of recombination on SNP distance estimation. Pairwise SNP differences were then calculated based on the core SNP alignment to generate a SNP distance matrix, which was visualized as a heatmap.

## 3 Result

### 3.1 Bacterial isolates and clinical characteristics

Among the 102 patients, four isolates belonging to non-*baumannii* members of the *Acinetobacter calcoaceticus–baumannii* (Acb) complex were excluded. Consequently, a total of 98 *A. baumannii* isolates collected between 2013 and 2021 were included in this study. The clinical characteristics of these patients are summarized in Table 1.

**Table 1:** Clinical characteristics.

Variable	*n* (%)
Basic demographics	
Age, years, mean ± SD	60.14 ± 29.70
Male	60 (61.2)
Location at time of culture	
Intensive Care Medicine (ICU)	43 (43.88)
Respiratory Medicine (PCCM)	14 (14.29)
General outpatient clinic	8 (8.16)
Neonatology	7 (7.14)
Burns clinic	6 (6.12)
Emergency department	4 (4.08)
Otolaryngology	2 (2.04)
Endocrinology	2 (2.04)
Gastroenterology	2 (2.04)
Cardiology	2 (2.04)
Thoracic surgery	2 (2.04)
Rheumatology	1 (1.02)
Bone oncology	1 (1.02)
Lymphoma department	1 (1.02)
Neurosurgery	1 (1.02)
Nephrology	1 (1.02)
Kidney transplant unit	1 (1.02)
Infection source	
Sputum	55 (56.12)
Bronchial lavage fluid	15 (15.31)
blood	6 (6.12)
Wound secretions	6 (6.12)
urine	5 (5.10)
Alveolar lavage fluid	3 (3.06)
Spinal fluid	1 (1.02)
Cerebrospinal fluid	1 (1.02)
Pus	1 (1.02)
Surgical excised tissue	1 (1.02)
Pleural effusion	1 (1.02)
Drainage fluid	1 (1.02)
Central venous cannula	1 (1.02)
Other specimens	1 (1.02)

The mean±SD age of patients with *A. baumannii* infection was 60.14 ± 29.70 (0–100 years), with an absolute male predominance of 61.2% (*n* = 60). The *A. baumannii* strains were mainly derived from sputum specimens (55 strains, 56.12%), Bronchial lavage fluid (15 strains, 15.31%) and blood specimens (6 strains, 6.12%). Patients with *A. baumannii* infection were mainly located in intensive care units (ICUs, 43.88%), respiratory medicine units (PCCMs, 14.29%), and General Outpatient Clinic (8.16%).

### 3.2 Antimicrobial susceptibility

The antimicrobial susceptibility of *A. baumannii* to *β*-Lactams, Quinolones, Aminoglycosides and Sulfonamidesis shown in Table 2. The resistance rates of *A. baumannii* to imipenem and meropenem were 70.41 and 95.74%, respectively. Even the two compounds with the greatest inhibitory activity, SXT and GEN inhibited *A. baumannii* by only 56.12 and 41.84%, respectively. In contrast, 59.09–69.39% of CRKP strains were resistant to the other β-lactam antibiotics CRO (69.39%), SAM (67.35%), FEP (67.35%), TZP (64.89%), CAZ (64.29%) and CSL (59.09). The *A. baumannii* strains also exhibited high resistance rates to quinolones CIP (73.47%) and LVX (46.94%) and aminoglycosides AMK (67.35%) and TOB (57.14%).

**Table 2:** Antimicrobial susceptibility of AB to β-lactams, quinolones, aminoglycosides, and sulfonamides.

Antibiotic	Subclasses	*R* (%)	*I* (%)	*S* (%)
β-lactams	CRO	69.39 (68/98)	17.35 (17/98)	13.27 (13/98)
	CAZ	64.29 (63/98)	25.51 (25/98)	10.20 (10/98)
	FEP	67.35 (66/98)	30.61 (30/98)	2.04 (2/98)
	IPM	70.41 (69/98)	\	29.59 (29/98)
	MEM	95.74 (45/47)	\	4.26 (2/47)
	SAM	67.35 (66/98)	28.57 (28/98)	4.08 (4/98)
	TZP	64.89 (61/94)	8.51 (8/94)	26.60 (25/94)
	CSL	59.09 (52/88)	10.23 (9/88)	30.68 (27/88)
Quinolones	LVX	46.94 (46/98)	30.61 (30/98)	22.45 (22/98)
	CIP	73.47 (72/98)	\	26.53 (26/98)
Aminoglycosides	GEN	45.92 (45/98)	12.24 (12/98)	41.84 (41/98)
	TOB	57.14 (56/98)	39.80 (39/98)	3.06 (3/98)
	AMK	67.35 (33/49)	2.04 (1/49)	30.61 (15/49)
Sulfonamides	SXT	43.88(43/98)	\	56.12 (55/98)

### 3.3 From genomic sequencing to phylogenetic inference

#### 3.3.1 MLST typing results and plasmid identification

**Figure 2:**
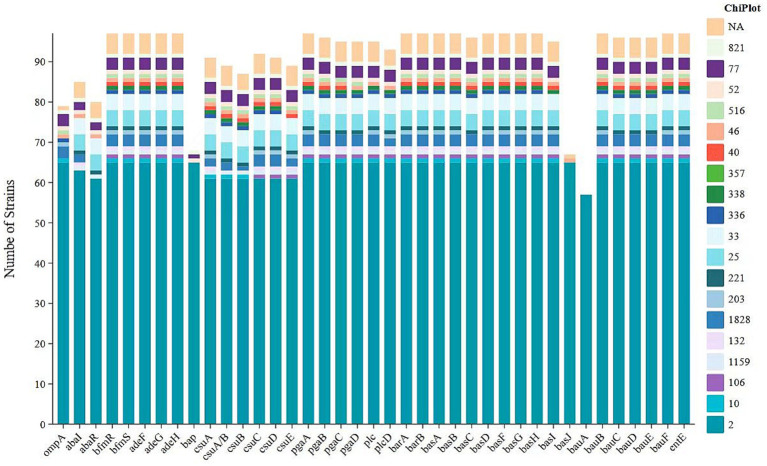
Variation in the distribution of virulence factors among *Acinetobacter baumannii* sequence types.

Based on the MLST analysis using the Pasteur scheme, the results are presented in Figure 2. Twenty ST types were identified among 98 strains of *A. baumannii*: ST2 (*n* = 65), ST25 (*n* = 4), ST33 (*n* = 4), ST77 (*n* = 3), ST1828 (*n* = 3), ST40 (*n* = 2) and other ST typing. No plasmids were found in 98 strains.

#### 3.3.2 Genomic profiling of antibiotic resistance

A total of 40 antibiotic resistance genes spanning eight classes were predicted by comparing the genomic data against the CARD database using ABRicate with default parameters. These include genes for sulfonamide (*sul1*, *sul2*), tetracyclines (*tet(A)*, *tet(B)*, *tet(39)*), phenicols (*catB8*、*cmlA1*), beta-lactams (*bla*_ADC-25_, *bla*_OXA-23_, *bla*_OXA-64_, *bla*_OXA-66_, *bla*_OXA-68_, *bla*_OXA-72_, *bla*_OXA-78_, *bla*_OXA-80_, *bla*_OXA-88_, *bla*_OXA-95_, *bla*_OXA-98_, *bla*_OXA-106_, *bla*_OXA-120_, *bla*_OXA-259_, *bla*_OXA-378_, *bla*_OXA-430_, *bla*_OXA-441_, *bla*_OXA-531_, *bla*_OXA-533_, *bla*_PER-7_, *bla*_TEM-1_), aminoglycosides (*aph*(3″)-Ib, *aph*(3′)-Ia, *aph*(6)-Id, *aac*(3)-IIa, *aac*(3)-Ia, *aac*(6′)-Ian, *aac*(6′)-Ib, *armA*, *aadA1*) and rifampicin (*aar-2*) resistance. Of the 98 strains analysed, one strain was found to possess a single resistance gene, 22 strains were found to possess two resistance genes, and the strain with the most genes had 16 resistance genes (Figure 1). ST2-type *A. baumannii* exhibits highly consistent multidrug resistance characteristics in both phenotypic and genotypic resistance patterns. It has been observed to demonstrate a high degree of resistance to a range of commonly prescribed antibiotics, including CRO, CAZ, FEP and CIP. In addition, the majority of strains manifest resistance to TZP and SAM. Genotypic analysis revealed that ST2 strains commonly harbor the *bla*_OXA-23_ carbapenemase gene, with high-frequency detection of aminoglycoside-modifying enzymes such as *aph*(3′)-Ia, *aph*(3″)-Ib, and *aph*(6)-Id, alongside sulfonamide and tetracycline resistance genes including *sul1*, *sul2*, and *tet(B)*. Furthermore, genes that mediate high-level aminoglycoside and macrolide resistance, such as *armA*, *mphE*, and *msrE*, are also commonly found in ST2 strains. Conversely, non-ST2 strains (e.g., ST33, ST77) demonstrate a greater diversity of resistance phenotypes, with certain strains maintaining susceptibility to multiple drugs and exhibiting lower rates of resistance genes.

Ten predicted resistance genes had a detection rate greater than 50%: *bla_ADC-25_* (91.84%, 90/98), *tet(B)* (71.43%, 70/98), *aph*(3″)-Ib (70.41%, 69/98), *aph*(6)-Id (70.41%, 69/98), *bla_OXA-23_* (67.35%, 66/98), *bla*_OXA-66_ (64.29%, 63/98), *bla*_TEM-1_ (60.20%, 59/98), *armA* (55.10%, 54/98), *msrE* (54.08%, 53/98) and *mphE* (53.06%, 52/98). Additionally, *sul2* also demonstrate high prediction rates, at 42.86% (42/98).

#### 3.3.3 Virulence determinants

The virulence potential of the 98 strains was assessed by screening their genomes against the Virulence Factor Database (VFDB) using ABRicate. This analysis identified 46 distinct virulence genes across the cohort. The gene repertoire per strain varied between 31 and 46. Functionally, these genes were categorized into five groups based on VFDB annotation, dominated by those involved in iron uptake (43.5%, 20/46), followed by biofilm formation (30.4%, 14/46), immune evasion (17.4%, 8/46), enzyme production (4.3%, 2/46), and regulation (4.3%, 2/46). As shown in Figure 2, significant differences were observed in the distribution of virulence factors among different sequence types (STs) of *Acinetobacter baumannii*, suggesting that certain STs may possess enhanced pathogenic potential. Notably, ST2 (represented by the blue segments in the figure) demonstrated a dominant presence across nearly all virulence factors, indicating a systematic enrichment of virulence-associated genes in this strain. Furthermore, the prevalence of ST2 was not sporadic but showed a persistent and stable trend, underscoring its strong capacity for colonization, transmission, and pathogenicity within hospital environments. In addition, the ST2 isolates in our cohort exhibited a marked enrichment of iron acquisition systems, indicating that iron uptake is an important determinant of their virulence. Specifically, all ST2 strains (65/65, 100%) carried the complete *Acinetobactin* biosynthesis gene cluster (*basA*–*basJ*) as well as the catecholate siderophore gene *entE*. The acinetobactin transport system (*bauB*–*bauF*) was conserved in all ST2 isolates, and the outer membrane receptor *bauA* was present in 57 of 65 strains (87.7%). This genetic configuration indicates that ST2 strains possess a largely intact and highly efficient siderophore-mediated iron acquisition network.

#### 3.3.4 The Pan-genome landscape of Clinical *A. baumannii* isolates

A pan-genomic analysis of 98 *A.baumannii* isolates was conducted using Roary with default parameters. A total of 11,723 genes were identified and classified into core (99% ≤ strains ≤ 98; 2,117 genes), soft core (95% ≤ strains < 99%; 462 genes), shell (15% ≤ strains < 95%; 1,533 genes), and cloud (strains < 15%; 7,611 genes) categories, with the latter two collectively defined as accessory genes.

**Figure 3:**
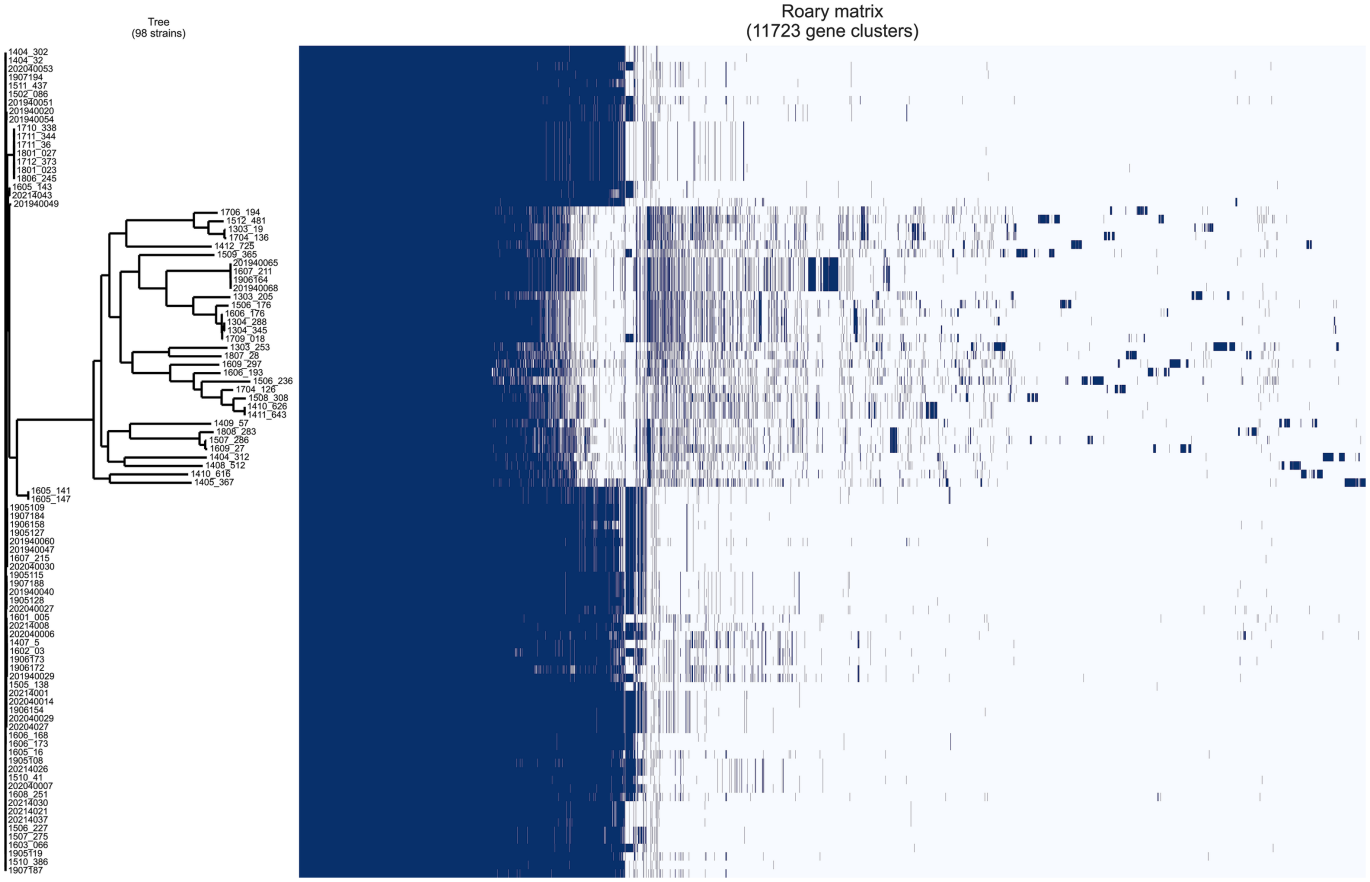
The results of pan-genomic analysis and evolutionary relationship analysis of *Acinetobacter baumannii* strains.

The pan-genome analysis and evolutionary relationship analysis results of Acinetobacter baumannii strains are shown in Figure 3.

These findings suggest a highly diverse accessory genome, which may contribute to the species’ remarkable adaptability to hospital environments and antibiotic pressure. The substantial proportion of accessory genes—particularly the large number of strain-specific cloud genes—likely reflects the acquisition of mobile genetic elements and genomic plasticity that underlie the success of this pathogen in nosocomial settings.

#### 3.3.5 Analysis of ST2 potential hospital transmission

**Figure 4:**
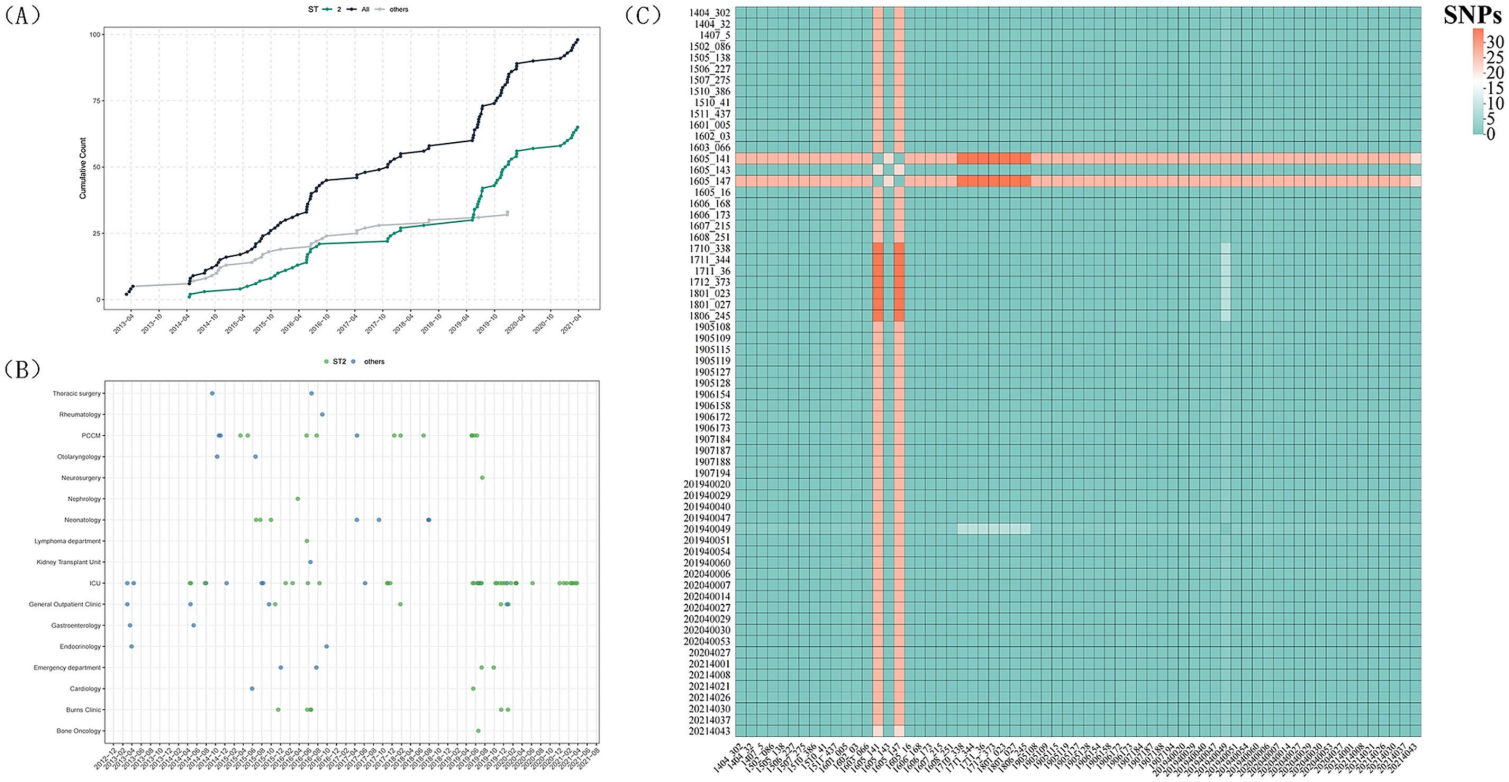
ST2 intrahospital transmission analysis. **(A)** Temporal accumulation of ST2 and non-ST2 isolates: cumulative numbers of ST2 (green curve), non-ST2 (grey line), and total *A. baumannii* isolates (grey line) collected between 2013 and 2021. **(B)** Departmental and temporal distribution of ST2 isolates and non-ST2 isolates. **(C)** Core-genome SNP distance heatmap of ST2 isolates.

To investigate the intrahospital transmission of ST2 *Acinetobacter baumannii*, a total of 65 ST2 isolates collected between 2014 and 2021 were analyzed (no ST2 isolates were detected in 2013). These isolates originated from multiple hospital departments, with a predominance in high-risk unitsICU-related units including the ICU and PCCM, as well as hematology and transplant wards (Figures 4A,B). The cumulative incidence curve revealed several marked stepwise increases over time, indicating episodic surges rather than sporadic independent cases.

Core-genome SNP analysis demonstrated that the ST2 isolates were highly conserved at the genomic level, with the vast majority of pairwise distances falling between 0 and 10 SNPs and a substantial proportion of isolates being genomically indistinguishable (0–2 SNPs) (Figure 4C). Using the commonly accepted threshold of ≤15 SNPs as evidence of recent transmission, nearly all ST2 isolates met this criterion despite being collected over a seven-year period, indicating prolonged persistence and recurrent expansion of a single epidemic clone rather than repeated independent introductions. The SNP heatmap further revealed large, homogeneous blocks consistent with a highly monophyletic population, with only minor sublineages showing slightly higher divergence (approximately 20–30 SNPs).

Integration of genomic and spatiotemporal clinical data identified three distinct outbreak periods in the ICU (June–August 2019, December 2019–June 2020, and December 2020–April 2021), during which multiple nearly identical ST2 isolates were recovered, providing strong evidence of sustained intrahospital transmission driven by a highly adapted ST2 clone.

## 4 Discussion

*A. baumannii* is the most prevalent Gram-negative bacillus within the genus *Acinetobacter* and is an opportunistic pathogen (Humphries et al., 2021). In the most recent 2024 WHO BPPL, carbapenem-resistant *A. baumannii* continues to be classified as a critical priority pathogen, necessitating the acceleration of efforts to develop new drugs and implement control measures (Jesudason et al., 2024; Jachowicz-Matczak et al., 2025). For patients, treating CRAB infections represents a global challenge (Zhou et al., 2024). In the event of the current limited treatment options proving unsuccessful, CRAB infections have the potential to be catastrophic for patients (Eslami et al., 2025). The present study observed a significantly higher incidence of *A. baumannii* infection in males than in females, consistent with previous reports (Uslan et al., 2007). Furthermore, a significant increase in infection rates was observed in ICUs in comparison to general wards (Tabah et al., 2023). This observation is reasonable, Patients in the ICU ward present with complex and critical conditions, often exhibiting compromised immunity, a history of multiple antimicrobial agents, and frequent invasive procedures. The treatment and recovery of these patients typically necessitates prolonged hospitalisation. The migration of *A. baumannii* from colonized sites is facilitated by these factors in combination with invasive interventions, thereby transforming it into a pathogenic bacterium. This phenomenon engenders conditions conducive to the proliferation of *A. baumannii* infections. Patients undergoing tracheal intubation/tracheotomy are often sedated, which can impair their ability to clear airway secretions and increase the risk of infection due to the downward migration of respiratory colonizing bacteria. Research has demonstrated that *A. baumannii* colonization rates are 68.1% in the nasal vestibule and 12.8% in the pharynx (Wang et al., 2012). In this study, 56.12% of isolates were obtained from sputum and 15.31% from bronchial lavage fluid, indicating that the respiratory tract is a common site of *A. baumannii* colonization and infection.

Molecular epidemiology is crucial for understanding the relatedness, evolutionary dynamics, and transmission of different *Acinetobacter baumannii* lineages, making MLST and whole-genome–based typing indispensable tools. Globally, ST1, ST2, ST25, and ST79 represent the major epidemic lineages of *A. baumannii* (Hamidian and Nigro, 2019), with ST2 being the dominant clone in China (Li et al., 2019). In the present study, ST2 was likewise identified as the predominant sequence type, consistent with large-scale national and international surveillance data (Duan et al., 2024; Fonseca et al., 2023). Recent phylogenomic analyses have further demonstrated that ST2 is not a sporadically emerging lineage, but rather originated in China in the mid-1970s and subsequently underwent multiple waves of clonal expansion in hospital settings, ultimately becoming a long-standing and persistently circulating dominant lineage (Zhang et al., 2025). In line with this evolutionary and epidemiological context, our analysis further revealed evidence of potential intrahospital transmission of ST2 *A. baumannii* in our center, characterized by tight genomic clustering and spatiotemporal aggregation of isolates. The high degree of genomic conservation (0–10 SNPs) among ST2 isolates across nearly a decade of sampling is striking. This pattern suggests that rather than undergoing rapid diversifying evolution, the ST2 population in our facility has undergone purifying selection, maintaining a highly optimized genomic architecture. The evolution of this clone appears to be characterized by the acquisition and subsequent fixation of modular resistance determinants (e.g., *bla*_OXA-23_, *armA*), leading to a stable, dominant ‘MDR backbone’ that is evolutionarily superior to sporadic non-ST2 lineages.

In China, the strong association between ST2 and carbapenem resistance has been systematically documented. A large-scale genomic study of 2,861 *A. baumannii* isolates collected in China between 1999 and 2022 showed that 96.9% of ST2 strains carried the acquired carbapenemase gene *bla*_OXA-23_ and 98.9% harbored the intrinsic *bla*_OXA-66_, whereas the prevalence of these two genes among non-ST2 isolates was only 34.3 and 13.4%, respectively (Zhang et al., 2025). These national-level data are highly consistent with our findings: in our ST2 isolates, the detection rates of *bla*_OXA-23_ and *bla*_OXA-66_ reached 93.85 and 96.92%, respectively, and the carbapenem resistance rate of ST2 was as high as 95.38%, markedly exceeding that observed in other sequence types. Further genotypic analysis revealed that ST2 strains not only stably carry OXA-type carbapenemases but also systematically accumulate multiple additional resistance determinants, including the aminoglycoside-modifying enzymes *aph*(3′)-Ia, *aph*(3′′)-Ib and *aph*(6)-Id, the sulfonamide and tetracycline resistance genes *sul1, sul2* and *tet(B)*, as well as genes mediating high-level aminoglycoside and macrolide resistance such as armA, mphE and msrE. This “modular resistance gene combination” indicates that ST2 has evolved into a highly stable genetic background carrying clustered multidrug-resistance determinants. In contrast, non-ST2 lineages (e.g., ST33 and ST77) display much greater phenotypic and genotypic heterogeneity, with some strains remaining susceptible to multiple antibiotics and exhibiting substantially lower rates of resistance gene carriage. Notably, large-scale genomic studies have shown that the dominance of ST2 is not merely the result of antibiotic selection pressure, but instead reflects a broader evolutionary strategy characterized by extensive recombination and continuous optimization of resistance- and virulence-associated loci, including surface polysaccharides and iron acquisition systems (Liu et al., 2022; Tascini et al., 2025). This evolutionary plasticity allows ST2 to maintain high-level carbapenem resistance while simultaneously enhancing its fitness, persistence, and transmissibility in hospital environments. In this context, the consistently high prevalence of carbapenem resistance (>85%) reported for ST2 worldwide (Duan et al., 2024; Gu et al., 2024) should be interpreted as the outcome of sustained clonal expansion of a biologically optimized, outbreak-prone lineage, rather than independent emergence of resistance in multiple genetic backgrounds.

In addition to its close association with carbapenem resistance, ST2 strains are also recognized for their elevated virulence. Studies have shown that ST2 isolates frequently harbor an extensive arsenal of virulence factors, including genes involved in biofilm formation, iron acquisition systems (such as those encoding siderophores like acinetobactin), and secretion systems, which enhance their ability to survive in hostile environments, evade host immunity, and establish persistent infections (Lurie-Weinberger et al., 2025). This combination of high resistance and enhanced virulence likely contributes to the successful dissemination and poor clinical outcomes associated with ST2 *A. baumannii* clones. Consistent with this, our study revealed a marked enrichment of iron acquisition genes in ST2 *Acinetobacter baumannii*. In particular, the complete acinetobactin biosynthesis cluster (*basA*–*basJ*) and the transport system (*bauB*–*bauF*) were universally conserved, *entE* was present in all isolates, and the outer membrane receptor *bauA* was detected in 87.7% of ST2 strains, indicating a highly intact and efficient siderophore-mediated iron uptake system. Because iron is strictly limited in the host, iron acquisition is a key determinant of *A. baumannii* virulence, and acinetobactin has been shown to be essential for *in vivo* survival and pathogenicity (Yehya et al., 2025). This optimized iron-scavenging capacity likely promotes bacterial fitness in hospital and host environments, providing a mechanistic basis for the tight SNP clustering, outbreak-prone behavior, and poor clinical outcomes associated with ST2 clones. Importantly, the strong dependence of ST2 on siderophore-mediated iron uptake also highlights these systems as promising therapeutic targets, both through direct inhibition of metallophore pathways and through Trojan-horse antibiotic strategies that exploit siderophore receptors to deliver drugs into multidrug-resistant cells (Ezzeddine and Ghssein, 2023; Tillotson, 2016).

All CRAB strains in this study were multidrug-resistant, with the majority belonging to the highly virulent ST2 clone. In contrast, non-MDR strains exhibited greater genetic diversity (Chen et al., 2024). Pan-genome analysis further highlighted the genomic plasticity of the isolates, with accessory genomes (including shell and cloud genes) accounting for 76.4% of the total gene pool—significantly larger than the core genome (23.6%). This suggests a high degree of genetic mobility and adaptive potential, often associated with the acquisition of antibiotic resistance genes, virulence determinants, and mobile genetic elements. Such genomic flexibility may underpin the successful expansion and persistence of ST2 strains in hospital settings in Guangzhou (Galac et al., 2020; Milani et al., 2021). The open pan-genome structure of *A. baumannii*, continuously integrating new accessory genes, likely facilitates its adaptation to diverse clinical environments (Ali et al., 2022). The convergence of genomic plasticity and the multidrug-resistant phenotype observed in this study emphasizes the need for strengthened surveillance and control measures against high-risk clones in healthcare settings.

This was a single-center retrospective study, which may limit the generalizability of the observed epidemiological and genomic patterns of *A. baumannii*. In addition, because no ST2 isolates were available from 2013, the earliest phase of ST2 introduction into our hospital could not be directly evaluated. Although strong genomic clustering and ward-level aggregation of ST2 isolates were identified, the absence of environmental and healthcare worker sampling prevented precise reconstruction of transmission routes. Moreover, the use of short-read sequencing and database-based annotation may have limited the detection of novel resistance determinants, plasmid-mediated transmission, and structural genomic variations. Future studies should therefore integrate multicenter prospective surveillance, long-read sequencing, environmental sampling, and real-time clinical metadata to better resolve ST2 transmission networks and support early detection and targeted infection control of high-risk clones.

## 5 Conclusion

In conclusion, this retrospective genomic epidemiological study provides a comprehensive characterisation of *Acinetobacter baumannii* clinical isolates from a tertiary hospital in Guangzhou over a nine-year period. The findings of this study serve to emphasise the ongoing predominance of the ST2 clone, which demonstrated elevated levels of carbapenem resistance and an extensive array of antimicrobial resistance genes, including *bla*_OXA-23_ and *bla*_OXA-66_. These factors are likely to have contributed to its effective nosocomial propagation. The high prevalence of resistance to multiple antibiotic classes is indicative of the critical challenge posed by CRAB in clinical settings. Furthermore, the pan-genome analysis revealed a highly plastic accessory genome, facilitating rapid adaptation and resistance acquisition. The prevalence of virulence factors associated with iron acquisition and biofilm formation further elucidates the pathogenic potential of these isolates. These results emphasise the urgent need for strengthened infection control measures, antimicrobial stewardship programs, and continuous genomic surveillance to monitor the evolution and spread of high-risk clones. It is recommended that future studies concentrate on the functional validation of resistance and virulence mechanisms, as well as the role of mobile genetic elements in the horizontal transfer of resistance genes. The objective of this research is to inform targeted therapeutic and preventive strategies.

## Data Availability

The datasets presented in this study can be found in online repositories. The names of the repository/repositories and accession number(s) can be found below: http://resolve.pid21.cn/13913.11.micro.data.project.NMDC10019931, NMDC10019931.

## Footnotes

1
https://card.mcmaster.ca/analyze

2
http://www.mgc.ac.cn/VFs/main.htm

3
http://www.mgc.ac.cn/VFs/
