# Supplementary material for: Whole-genome sequencing identifies persistent transmission of a high-risk ST2 Acinetobacter baumannii clone in a Guangzhou Hospital
Source: Front Microbiol. 2026 Feb 18;17:1730485. doi: 10.3389/fmicb.2026.1730485 (PMC12957087; doi:10.3389/fmicb.2026.1730485)
Supplement: Supplementary file 1 [file Table_1.docx]

Supplementary Material

Table S1 Information of patients

| Assembly | SEX | AGE | SPEC_DATE | SPEC_TYPE | WARDNAME |
| --- | --- | --- | --- | --- | --- |
| 1905108 | f | 79 | 2019/5/8 | sp | PCCM |
| 1905109 | m | 83 | 2019/5/8 | ba | PCCM |
| 1905115 | m | 69 | 2019/5/13 | sp | ICU |
| 1905119 | m | 65 | 2019/5/17 | sp | Cardiology |
| 1905127 | f | 39 | 2019/5/23 | sp | ICU |
| 1905128 | f | 77 | 2019/5/23 | sp | PCCM |
| 1906154 | m | 75 | 2019/6/10 | ba | PCCM |
| 1906158 | f | 23 | 2019/6/13 | ba | ICU |
| 1906164 | f | 3 | 2019/6/18 | sp | ICU |
| 1906172 | f | 24 | 2019/6/19 | wd | Bone Oncology |
| 1906173 | f | 68 | 2019/6/25 | sp | ICU |
| 1907184 | f | 85 | 2019/7/4 | sp | ICU |
| 1907187 | m | 84 | 2019/7/11 | sp | Emergency department |
| 1907188 | m | 47 | 2019/7/11 | sp | ICU |
| 1907194 | f | 45 | 2019/7/15 | sf | Neurosurgery |
| 20204027 | m | 66 | 2020/12/3 | sp | ICU |
| 20214001 | m | 73 | 2020/12/30 | sp | ICU |
| 20214008 | m | 65 | 2021/1/18 | ba | ICU |
| 20214021 | f | 73 | 2021/2/13 | sp | ICU |
| 20214026 | m | 84 | 2021/2/20 | sp | ICU |
| 20214030 | f | 73 | 2021/2/28 | sp | ICU |
| 20214037 | m | 15 | 2021/3/16 | ba | ICU |
| 20214043 | f | 58 | 2021/3/26 | sp | ICU |
| 201940020 | f | 52 | 2019/9/28 | ti | Emergency department |
| 201940029 | m | 44 | 2019/10/10 | su | ICU |
| 201940040 | m | 89 | 2019/10/19 | sp | ICU |
| 201940047 | f | 66 | 2019/11/7 | sp | ICU |
| 201940049 | f | 86 | 2019/11/14 | ur | General Outpatient Clinic |
| 201940051 | m | 35 | 2019/11/16 | wd | Burns Clinic |
| 201940054 | f | 40 | 2019/11/23 | sp | ICU |
| 201940060 | m | 77 | 2019/12/9 | sp | ICU |
| 201940065 | m | 88 | 2019/12/23 | ba | General Outpatient Clinic |
| 201940068 | m | 5 | 2019/12/24 | ba | ICU |
| 202040006 | f | 84 | 2019/12/30 | wd | Burns Clinic |
| 202040007 | f | 87 | 2020/1/2 | sp | General Outpatient Clinic |
| 202040014 | m | 100 | 2020/1/19 | ba | ICU |
| 202040027 | m | 85 | 2020/2/19 | sp | ICU |
| 202040029 | m | 95 | 2020/2/23 | sp | ICU |
| 202040030 | f | 85 | 2020/2/22 | wd | ICU |
| 202040053 | f | 57 | 2020/6/8 | sp | ICU |
| 1303_19 | f | 61 | 2013/3/2 | sp | ICU |
| 1303_205 | m | 86 | 2013/3/2 | sp | General Outpatient Clinic |
| 1303_253 | m | 83 | 2013/3/20 | bl | Gastroenterology |
| 1304_288 | f | 84 | 2013/3/30 | bl | Endocrinology |
| 1304_345 | m | 54 | 2013/4/13 | sp | ICU |
| 1404_302 | m | 1 | 2014/4/16 | sp | ICU |
| 1404_312 | m | 96 | 2014/4/20 | sp | General Outpatient Clinic |
| 1404_32 | m | 9 | 2014/4/23 | sp | ICU |
| 1405_367 | m | 84 | 2014/5/11 | sp | Gastroenterology |
| 1407_5 | f | 99 | 2014/7/24 | sp | ICU |
| 1408_512 | m | 0 | 2014/7/31 | sp | ICU |
| 1409_57 | f | 78 | 2014/9/10 | ot | Thoracic surgery |
| 1410_616 | f | 48 | 2014/10/12 | ps | Otolaryngology |
| 1410_626 | m | 83 | 2014/10/23 | ur | PCCM |
| 1411_643 | m | 83 | 2014/11/3 | ur | PCCM |
| 1412_725 | m | 73 | 2014/12/13 | sp | ICU |
| 1502_086 | m | 67 | 2015/3/14 | sp | PCCM |
| 1505_138 | f | 61 | 2015/4/30 | sp | PCCM |
| 1506_176 | m | 51 | 2015/5/29 | ba | Cardiology |
| 1506_227 | m | 1 | 2015/6/24 |  | Neonatology |
| 1506_236 | m | 65 | 2015/6/20 | bl | Otolaryngology |
| 1507_275 | m | 1 | 2015/7/21 | sp | Neonatology |
| 1507_286 | f | 16 | 2015/7/31 | sp | ICU |
| 1508_308 | m | 53 | 2015/8/9 | sp | ICU |
| 1509_365 | m | 99 | 2015/9/17 | sp | General Outpatient Clinic |
| 1510_386 | m | 89 | 2015/9/29 | sp | General Outpatient Clinic |
| 1510_41 | m | 0 | 2015/10/27 | sp | Neonatology |
| 1511_437 | m | 30 | 2015/11/16 | wd | Burns Clinic |
| 1512_481 | m | 76 | 2015/12/3 | bl | Emergency department |
| 1601_005 | m | 31 | 2016/1/4 | sp | ICU |
| 1602_03 | m | 63 | 2016/2/18 | cc | ICU |
| 1603_066 | f | 84 | 2016/3/23 | ur | Nephrology |
| 1605_141 | f | 87 | 2016/5/20 | wd | PCCM |
| 1605_143 | m | 56 | 2016/5/22 | sp | Lymphoma department |
| 1605_147 | f | 84 | 2016/5/24 | ba | Burns Clinic |
| 1605_16 | f | 85 | 2016/5/28 | sp | ICU |
| 1606_168 | f | 76 | 2016/6/14 | ba | Burns Clinic |
| 1606_173 | f | 76 | 2016/6/18 | ba | Burns Clinic |
| 1606_176 | m | 26 | 2016/6/14 | ur | Kidney Transplant Unit |
| 1606_193 | m | 56 | 2016/6/21 | pf | Thoracic surgery |
| 1607_211 | m | 74 | 2016/7/23 | sp | Emergency department |
| 1607_215 | f | 66 | 2016/7/25 | sp | PCCM |
| 1608_251 | m | 66 | 2016/8/12 | ba | ICU |
| 1609_27 | m | 70 | 2016/8/31 | bl | Rheumatology |
| 1609_297 | f | 62 | 2016/9/28 | bl | Endocrinology |
| 1704_126 | m | 0 | 2017/4/14 | sp | Neonatology |
| 1704_136 | m | 94 | 2017/4/15 | sp | PCCM |
| 1706_194 | m | 0 | 2017/6/7 | sp | ICU |
| 1709_018 | f | 0 | 2017/9/6 | sp | Neonatology |
| 1710_338 | m | 77 | 2017/10/30 | ba | ICU |
| 1711_344 | m | 77 | 2017/11/4 | sp | ICU |
| 1711_36 | m | 77 | 2017/11/19 | sp | ICU |
| 1712_373 | m | 77 | 2017/12/15 | ba | PCCM |
| 1801_023 | m | 77 | 2018/1/24 | sp | General Outpatient Clinic |
| 1801_027 | m | 78 | 2018/1/26 | ba | PCCM |
| 1806_245 | m | 96 | 2018/6/25 | ba | PCCM |
| 1807_28 | f | 0 | 2018/7/27 |  | Neonatology |
| 1808_283 | f | 0 | 2018/7/31 | sp | Neonatology |
